# Disparities in Elective and Emergency Caesarean Section Rates Among Public and Private Hospitals in the Districts of Andhra Pradesh, India

**DOI:** 10.7759/cureus.54320

**Published:** 2024-02-16

**Authors:** Nagendra Gavvala, Benson Thomas M, Gladius Jennifer H

**Affiliations:** 1 School of Public Health, SRM Institute of Science and Technology (SRMIST), Chennai, IND

**Keywords:** andhra pradesh, public and private hospitals, elective and emergency, disparities, caesarean section rates

## Abstract

Background

In India, there has been a steady increase in the rate of caesarean section (C-section) deliveries over the past decade, rising from 17% during National Family Health Survey-4 (NFHS-4 (2015-16)) to as high as 21.5% during NFHS-5 (2019-21). Andhra Pradesh, India, is experiencing a particularly high rate of 42.4% as per NFHS-5, which is the highest among the states in the country. This study aims to investigate the prevalence of C-section deliveries across the districts of Andhra Pradesh and to identify the disparities in elective and emergency C-section rates among public and private hospitals in districts of Andhra Pradesh, India.

Methods

The study utilized secondary data from the NFHS-5 conducted by the International Institute for Population Sciences, Mumbai. A statistical software package was used to perform the analysis, while a quantum geographic information system​​​​​​​ (QGIS) was used to prepare a map. Descriptive statistics, bivariate analysis, and multivariate binary regression were used for statistical analysis.

Results

Significant variations in the prevalence of C-section deliveries were found across the districts in Andhra Pradesh. The prevalence ranged from 22.2% in Anantapur to 66% in Krishna. It was also found that private hospitals were the primary drivers of the high prevalence of C-section deliveries. Approximately 31.51% of women underwent C-sections in public institutions, whereas it was 68.49% in private institutions. The overall occurrence of C-section deliveries in Andhra Pradesh was 65% for elective cases and 35% for emergency cases, indicating a relatively higher prevalence for elective procedures.

Conclusion

The choice of the medical institution, whether private or public, is the most significant factor influencing the high prevalence of C-section deliveries. Additionally, C-section deliveries were found with higher complication rates than normal deliveries. Elective C-sections are more prevalent in the state, and factors such as wealth quintile and birth order are impacting the likelihood of elective versus emergency C-section deliveries. The study suggests that the government should provide awareness and regulations to promote vaginal deliveries and prevent unnecessary C-sections in hospitals.

## Introduction

A caesarean section (C-section) is a necessary procedure in cases where there is a medical justification to prevent maternal and perinatal mortality and morbidity. However, like any surgery, it carries both short- and long-term risks that can affect the health of the woman, her child, and future pregnancies beyond the current delivery [1]. The World Health Organization (WHO) recommends that this procedure should only be used in cases of complicated pregnancies. In recent decades, there has been a significant increase in the number of C-section deliveries in both developed and developing countries [2-5]. Remarkably, the utilization rates of C-sections above 15% are inappropriate and can lead to unnecessary financial burdens and clinical risks for patients and healthcare systems [6-7].

National-level surveys in India show that the prevalence of C-section deliveries is on the increase over a period of time where it increases from 17% to 21.5% between the National Family Health Survey (NFHS-4) and NFHS-5 rounds. High prevalence is noticeable in the southern states of India, including 60.7% in Telangana, 44.9% in Tamil Nadu, 42.4% in Andhra Pradesh, 38.9% in Kerala, and 31.5% in Karnataka, as well as in the northern states such as Punjab with 38.5% and Jammu and Kashmir with 41.7%.

The increasing use of C-sections can be attributed to the rise in institutional deliveries. However, a study by Kumar et al. suggests that the type of healthcare facility (private or public) may be the strongest factor influencing C-section deliveries [8]. In addition, Desai et al. presented that financial incentives for doctors particularly in the private healthcare sector, avoidance of long hours of labor pain, along with the availability and affordability of medical facilities, as well as decreasing family size, etc., are some of the other noted factors [9].

In Andhra Pradesh, many women prefer a surgical delivery due to their unwillingness to bear the pain associated with labor, making it challenging to convince them to opt for natural birth [10]. Furthermore, the demand for C-sections is driven by cultural factors, such as the preference for auspicious dates and times for delivery. In such an environment, doctors may find it difficult to refuse a planned C-section, even when it may not be medically necessary [11]. Previously, complications during labor led to surgical interventions, but now scheduled surgeries have become more common, adding to the rise in C-section rates.

This study aims to estimate the prevalence and distribution of elective and emergency C-section deliveries in both public and private healthcare facilities in Andhra Pradesh. It also examines the role of various socio-demographic factors in determining C-section deliveries in the state.

## Materials and methods

Data source

The study uses secondary data collected from the NFHS-5 survey data for analytical purposes. The NFHS-5 is an extensive, multi-round survey carried out by the International Institute for Population Sciences (IIPS), Mumbai, Government of India, in a sample of households that represent the nation, encompassing approximately 99% of the Indian population. All the survey data were shared by IIPS Mumbai on submission of a formal written request over mail. A total of 2,734 cases were included who had a delivery (normal or C-section) in the two years preceding the survey either at public or private hospitals.

Inclusion criteria

NFHS-5 (2019-21) collected the data from women who delivered by normal or C-section (live birth and/or stillbirth) in the two years preceding the survey either at public or private hospitals.

Exclusion criteria

Women who delivered by normal or C-section (live birth and/or stillbirth) more than two years preceding the survey and women who had abortions were excluded from participating in the NFHS 5 (2019-21) survey.

Statistical analysis

The statistical analysis is mainly focused on the estimation of the levels and trends of C-section deliveries in Andhra Pradesh, as well as the disparities in the prevalence of C-sections across the districts. Descriptive statistics, bivariate analysis, and multivariate binary regression were used for statistical analysis. The t-test was used to compare the mean prevalence of women who underwent c-section deliveries in private and public hospitals. All the analyses were performed by using the Stata (StataCorp LLC, College Station, Texas) statistical software and QGIS (QGIS Development Team, Johannesburg, South Africa) software.

Description of dependent variables

The study examined the prevalence of C-section deliveries across the districts of Andhra Pradesh from 2019 to 2021. This analysis further extended to estimate the prevalence of C-sections by categorizing it by type of facility (government/private) or choice category (elective/emergency). The NFHS-5 data specify the status of C-section delivery by a question that has a binary response as ‘yes’ and ‘no’. Similarly, information about the 'place of delivery' was generated as 'public' and 'private' from a question that asks about the location of childbirth. Further, the choice of C-section delivery was categorized as 'elective' and 'emergency' based on whether the decision to have C-section delivery was made before or after the onset of labor pain.

Identified independent variables for the study

The study considered several variables, including place of residence (urban or rural), religion (Hindu, Muslim, or Christian), caste/sub-group (scheduled caste, scheduled tribe, other backward caste, or others), and wealth index (poorest, poorer, middle, richer, or richest). In the NFHS-5 survey, the wealth index is calculated using easy-to-collect data on a household's ownership of selected assets, such as televisions and bicycles; materials used for housing construction; and types of water access and sanitation facilities.

Other variables considered for the study include education level of the mother (no education, primary, secondary, or higher), birth order (1, 2, 3, or above), multiple births (single child or more than one child), complications during delivery (no or yes), antenatal care (ANC) visits (less than four or four and above), birth weight of child (low, medium, high, or don't know), maternal age during delivery (20 years and below, 21-30 years, or 31 years and above), and birth spacing (less than two years, 2-3 years, or more than three years).

The complications during delivery variable were created as a binary variable, with women experiencing any of the three complications during delivery (breech presentation of the baby, excessive bleeding of the mother, and prolonged labor) registering as one or, otherwise, zero.

## Results

**Figure 1:**
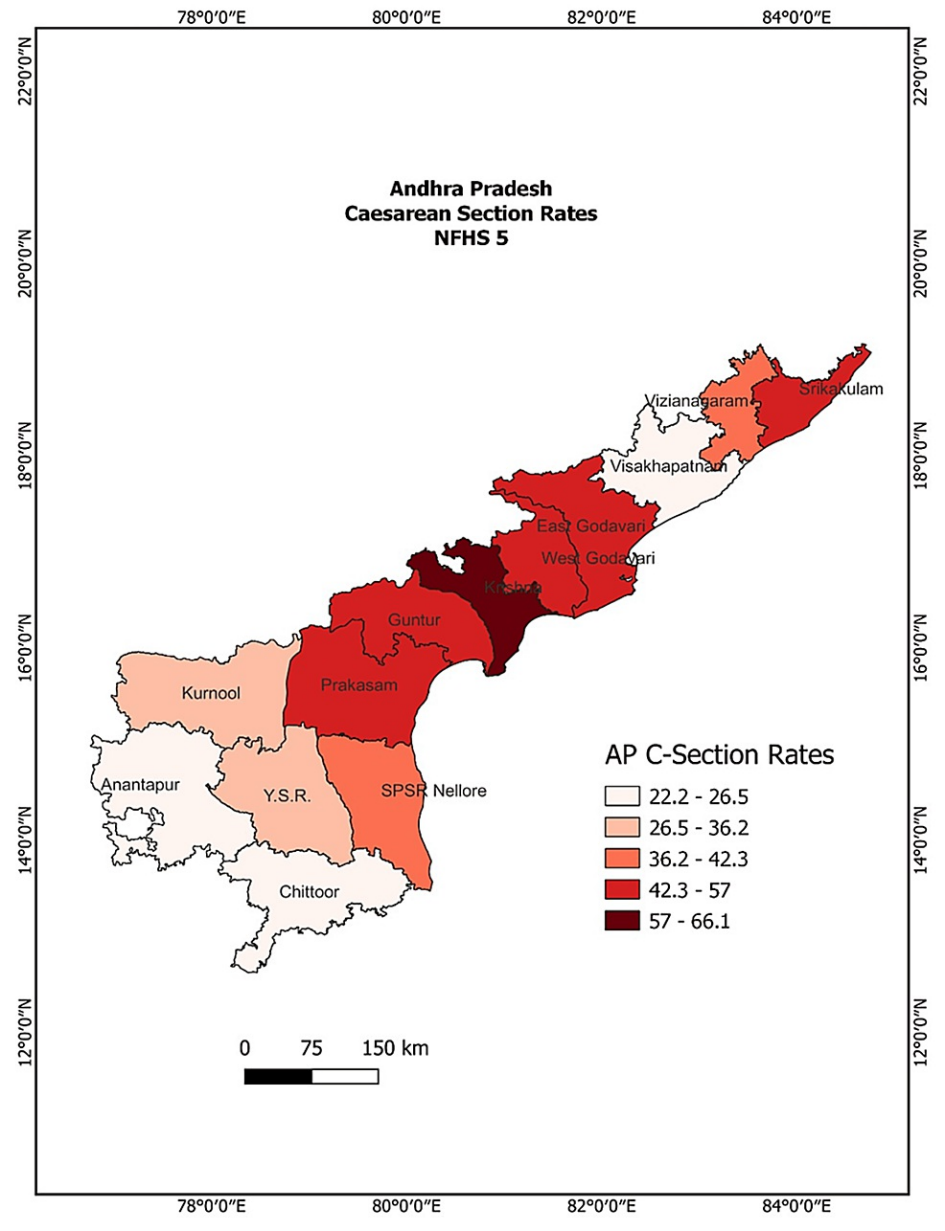
District-wise prevalence of caesarean section rates of Andhra Pradesh, classified as per NFHS-5 Reproduced using NFHS-5 district estimates of caesarean section rates of Andhra Pradesh using QGIS software.

Table 1 depicts the prevalence of C-section deliveries across the districts of Andhra Pradesh by type of facility and type of C-section. Figure 1 also shows the district-wise prevalence of C-section deliveries as per NFHS 5 (2019-21).

**Table 1:** Prevalence of caesarean deliveries among the districts of Andhra Pradesh during 2019-21 Source: Author’s estimation from NFHS-5 Survey Data

District	Delivery by caesarean section (%)	Place of caesarean section delivery		Type of caesarean delivery	
		Public	Private	Elective	Emergency
Srikakulam	57.0	40.8	59.2	71.0	29.1
Vizianagaram	41.3	51.7	48.3	72.3	27.7
Visakhapatnam	26.5	44.0	56.0	61.1	38.9
East Godavari	52.2	30.1	69.9	70.8	29.2
West Godavari	55.7	35.4	64.6	66.4	33.6
Krishna	66.1	29.1	70.9	58.2	41.8
Guntur	53.8	34.2	65.9	64.5	35.5
Prakasam	54.2	10.6	89.4	74.1	25.9
SPSR Nellore	42.3	15.5	84.5	54.4	45.6
Y.S.R.	36.2	29.5	70.5	58.5	41.5
Kurnool	32.9	26.7	73.4	70.5	29.5
Anantapur	22.2	42.6	57.4	68.9	31.1
Chittoor	26.4	36.7	63.3	58.9	41.1
Andhra Pradesh	42.4	31.5	68.5	65.3	34.7

The overall prevalence estimates show high variation across different districts ranging from 22.2% to 66.0%. The district Anantapur (22.2%) has the lowest prevalence, followed by Chittor (26.4%) and Visakhapatnam (26.5%), while Krishna district has the highest prevalence with 66.1%, followed by Srikakulam (57%), West Godavari (55.7%), and Prakasam (54%) in the state. Table 1 also shows the disparities in the prevalence of C-section deliveries and the place of delivery (public vs private healthcare institutions) across the districts. The prevalence of C-section deliveries is higher among the women who delivered in private healthcare institutions (68.49%) than that of their counterparts from public healthcare institutions (31.51%).

The pattern of the high prevalence of C-section deliveries among private healthcare institutions than that of public ones is evident across the districts. Among the districts, the prevalence of C-section deliveries in public healthcare institutions was the highest in Vizianagaram (52%), Visakhapatnam (44%), Anantapur (43%), Srikakulam (41%), and Chittoor (37%), followed by West Godavari (35%). On the other hand, the prevalence of C-section deliveries in public healthcare institutions was found to be the lowest in Prakasam (11%), Nellore (15%), and Kurnool (26%). Similarly, in Prakasam, 89% of women opted for private healthcare institutions, followed by 84% in Nellore. In Krishna, Y.S.R, and Kurnool districts, a significant proportion of women chose private healthcare facilities for C-section delivery.

Table 1 additionally exhibits the district-specific rates of C-section deliveries classified according to the type of C-section procedure (elective and emergency) in the Indian state of Andhra Pradesh. The findings indicate that the overall occurrence of C-section deliveries in Andhra Pradesh was 65% for elective cases and 35% for emergency cases, indicating a relatively higher prevalence for elective procedures. Nevertheless, there were notable disparities in the prevalence of C-section deliveries by delivery type across different districts within the state.

In Prakasam, the prevalence of elective C-section deliveries was the highest at 74%, followed by Vizianagaram (72%) and Srikakulam, East Godavari, and Kurnool at 71% for elective cases. In contrast, the prevalence estimate was the lowest in the same districts for emergency cases. In Visakhapatnam, around 39% of women underwent C-section deliveries due to emergencies, while the prevalence estimate for women undergoing C-sections with pre-planned processes was as high as 61%.

Table 2 displays the prevalence estimates of C-section deliveries in Andhra Pradesh, India, categorized by various demographic characteristics. The data indicate that urban women had the highest prevalence of C-section deliveries at 50.47%, followed by women from the Muslim community (43%) and other caste categories (53.24%). Additionally, there was a noticeable trend of increased likelihood of undergoing a C-section with higher levels of education and wealth quintile. For instance, approximately 45% of women in the richer wealth quintile and 61% of women in the wealthiest wealth quintile had experienced a C-section delivery. The prevalence estimates for C-section deliveries were also observed to rise with an increase in both education level and wealth quintile.

**Table 2:** Association between the prevalence of caesarean section and socio-economic and demographic characteristics in Andhra Pradesh, India (NFHS-5 (2019-21) OR: Odds ratio; CI: Confidence interval; ®: Reference category *, **, and *** refer to < 0.05, < 0.01, and < 0.001 levels of significance, respectively.

Background characteristics	Prevalence	OR	CI	P-value
Place of Residence				
Urban®	50.47	1 [Reference]		
Rural	39.28	0.634***	[0.573-0.703]	0.000
Religion				
Hindu®	42.76	1 [Reference]		
Muslim	43.54	1.032	[0.874-1.218]	0.708
Christian	39.13	0.86	[0.738-1.003]	0.055
Caste				
Schedule Caste®	36.69	1 [Reference]		
Schedule Tribe	21.71	0.478***	[0.373-0.613]	0.000
OBC	43.88	1.348***	[1.202-1.512]	0.000
Others	53.24	1.964***	[1.702-2.267]	0.000
Wealth Index				
Poorest®	18.48	1 [Reference]		
Poorer	31.55	2.033***	[1.534-2.695]	0.000
Middle	42.64	3.280***	[2.500-4.303]	0.000
Richer	44.93	3.600***	[2.738-4.734]	0.000
Richest	61.19	6.956***	[5.216-9.276]	0.000
Education level				
No education®	27.36	1 [Reference]		
primary	31.51	1.221	[0.992-1.503]	0.059
Secondary	42.96	2.000***	[1.710-2.338]	0.000
Higher	58.53	3.747***	[3.123-4.496]	0.000
Place of delivery				
Public®	26.6	1 [Reference]		
Private	63	4.712***	[4.259-5.213]	0.000
Birth order				
1®	45.14	1 [Reference]		
2	44.92	0.878*	[0.787-0.980]	0.020
3 and above	27.28	0.292***	[0.247-0.346]	0.000
Multiple Births				
Single Child®	48.6	1 [Reference]		
More than 1 child	40.41	0.694***	[0.622-0.773]	0.000
Complications during delivery				
No®	42.44	1 [Reference]		
Yes	41.47	0.961	[0.875,1.055]	0.405
ANC visits				
Less than 4®	41.22	1 [Reference]		
4 and above	48.06	1.325***	[1.188-1.476]	0.000
Size of a child at birth				
Low®	36.56	1 [Reference]		
Medium	41.34	1.222*	[1.027-1.453]	0.023
High	45.87	1.494***	[1.246-1.792]	0.000
Don't know	19.23	0.413	[0.154-1.107]	0.079

Interestingly, the data reveal that more than 63% of women in Andhra Pradesh opted for private healthcare institutions for their delivery, where the prevalence of C-section deliveries was also higher. Women with a birth order of one or two also had a higher likelihood of undergoing a C-section delivery, with prevalence estimates of 45% and 44.9%, respectively. C-sections were also commonly observed among women having a single child (48.6%), those who had four or more ANC visits (48%), and those giving birth to a larger child (46%). It is worth noting that the prevalence of C-section deliveries was marginally higher among women who did not face complications during delivery (42.4%) compared to those who did (41.4%).

Table 2 further showcases the outcomes of regression models investigating the connections between C-section deliveries and diverse covariates among married women in Andhra Pradesh, India. The examination discloses that several variables, including the woman's residential location, caste, wealth index, educational attainment, delivery location, birth order, the occurrence of multiple births, count of ANC visits, and the size of the child at birth, exhibit significant associations with the probability of experiencing a C-section.

The bivariate analysis and multivariate binary regression results reveal several factors associated with the likelihood of having a C-section. According to the findings, women residing in rural areas (adjusted odds ratio (AOR): 0.63, 95% confidence interval (CI): 0.573-0.703), Christians (AOR: 0.86; CI: 0.738-1.003), and those belonging to scheduled tribes (AOR: 0.47, CI: (0.373-0.613) exhibit lower odds of undergoing a C-section compared to their counterparts. Conversely, Muslim women (AOR: 1.03, CI: 0.874-1.218) and those categorized under the 'other' caste (AOR: 1.96; CI: 1.702-2.267) are more likely to undergo a C-section. The choice of healthcare institution also influences the likelihood, as women delivering in private healthcare facilities show significantly greater odds of having a C-section (AOR: 4.71, CI: 4.259-5.213) compared to those delivering in public healthcare facilities.

Furthermore, the study identifies education level and wealth index as factors contributing to increased odds of having a C-section. Women with higher education levels and those from wealthier households demonstrate higher odds of undergoing a C-section. Birth order plays a role, with women having a third or higher order of birth showing a reduced likelihood of a C-section (AOR: 0.29, CI: 0.247-0.346). Women experiencing multiple births (AOR: 0.694, CI: 0.622-0.773) and those facing complications during pregnancy (AOR: 0.961, CI: 0.875, 1.055) are less likely to have a C-section. Conversely, women with four or more ANC visits (AOR: 1.32, CI: 1.188-1.476) and those delivering larger babies (AOR: 1.5, CI: 1.246-1.792) have significantly higher odds of undergoing a C-section.

Overall, the results highlight the complex interplay of various factors that contribute to the prevalence of C-section deliveries in Andhra Pradesh. The findings also underscore the need for targeted interventions to promote safe and appropriate delivery practices in the state.

Table 3 depicts the bivariate estimates of C-section deliveries, classified into public and private healthcare facilities, based on several background characteristics of the women. The data indicate that C-section deliveries are primarily performed in private healthcare institutions rather than public ones. The study observed that Christian women preferred public health facilities for C-sections, whereas more Muslim women chose private health facilities for this purpose. In terms of caste, the prevalence of scheduled caste women undergoing C-sections in public health facilities was as high as 37%, while 78% of women from other castes sought private healthcare institutions for their C-section deliveries.

**Table 3:** Caesarean sectional deliveries in public-private healthcare institutions and type of caesarean section by selected background characteristics in Andhra Pradesh, India, NFHS-5 (2019-21)

Category	Variable	Sample frequency with caesarean section	Overall caesarean section rates (%)	Place of delivery		Type of caesarean delivery	
				Public	Private	Elective	Emergency
Age	<=24 yrs	472	38.61	37.35	62.65	62.01	37.54
	25-29 yrs	469	43.25	28.05	71.95	67.71	32.29
	>=30 yrs	242	50.25	27.05	72.95	66.36	33.64
Education	No education	99	27.36	43.37	56.63	67.49	32.51
	Primary	94	31.51	40.68	59.32	53.29	46.71
	Secondary	717	42.96	35.71	64.29	66.77	33.23
	Higher	273	58.53	13.93	86.07	64.96	35.04
Place of residence	Urban	358	50.47	25.56	74.44	66.38	33.62
	Rural	825	39.28	34.52	65.48	64.76	35.24
Religion	Hindu	976	42.76	32.44	67.56	65.55	34.45
	Muslim	104	43.54	20.97	79.03	65.75	34.25
	Christian	103	39.13	33.2	66.8	62.85	37.15
Caste	SC/ST	284	33.88	39.32	60.68	64.17	35.83
	OBC	657	43.88	31.89	68.11	65.04	34.96
	Others	242	53.24	22.1	77.9	67.15	32.85
Wealth index	Poorer	208	28.85	49.62	50.38	59.6	40.1
	Middle	398	42.64	36.82	63.18	65.89	34.11
	Richer	577	50.44	21.75	78.25	66.64	33.1
ANC visits	less than 4	245	41.22	35.14	64.86	63.01	36.99
	4 and above	683	48.06	30.36	69.64	67. 94	32.06
Place of C-section	Public	373	26.55	100	0	63.21	36.63
	Private	810	63.01	0	100	66.1	33.71
Complications during delivery	No	290	42.44	28.38	71.62	66.16	33.84
	Yes	635	41.47	35.30	64.70	63.6	36.4
	Data N/A	258	-	-	-	-	-
Birth order	1	554	45.14	31.15	68.85	59.54	40.46
	2	520	44.92	32.26	67.74	66.49	33.51
	3 and above	109	27.28	27.58	72.42	72.63	27.37
Multiple births	Single Child	641	48.6	28.46	71.54	66.16	33.84
	More than 1 child	542	40.41	32.65	67.35	61.33	38.67
Birth weight of child	Low	88	36.56	41.96	58.03	71.23	28.77
	Medium	675	41.34	31.91	68.09	65.54	34.46
	High	418	45.87	30.1	69.89	63.53	36.47
Andhra Pradesh		1183	21.44	31.53	68.47	65.31	34.69

Regarding the wealth index, the study found that women from the poorest wealth quintile (62%) had the highest proportion of C-section deliveries in public health facilities. On the other hand, women from the richest wealth quintile (85%) had the highest proportion of C-section deliveries in private healthcare institutions. In terms of education, 14% of women with higher education underwent C-sections in public health facilities, while 86% opted for private health facilities.

The study also observed that the likelihood of undergoing C-section deliveries in public health facilities decreased with an increase in the birth order. Around 31% of women with their first birth underwent C-sections in public health facilities, while only about 27% of women with third or higher birth preferred public health facilities. The study also found that women who had complications during delivery had a higher likelihood of undergoing C-section deliveries in private healthcare institutions. Almost 28% of women who faced complications during delivery opted for C-sections in public health facilities, while 72% chose private health facilities.

Overall, the findings suggest that the choice of healthcare facilities for C-section deliveries varies significantly based on several background characteristics, such as religion, caste, wealth index, education, birth order, and complications during delivery.

Table 3 also presents the prevalence of C-section delivery in Andhra Pradesh, categorized by selected background characteristics and type of C-section delivery. The data reveal that C-sections were more prevalent among urban women (66%) for elective pregnancy, while more rural women (35%) underwent C-sections in case of emergency.

The research also revealed notable variations in the prevalence of C-sections across different wealth quintiles. Approximately 65% of women in the wealthiest quintile opted for pre-planned C-sections, whereas 49% of women in the poorest quintile underwent emergency C-sections. Moreover, the data indicated that women with higher birth orders were more inclined towards elective C-sections. Specifically, 72.6% of women with a birth order of three or more underwent elective C-sections, whereas 40% of women with a birth order of one experienced emergency C-sections.

Furthermore, the study demonstrated significant differences in the prevalence of C-sections based on the presence of complications during delivery. Approximately 66% of women without complications chose elective C-sections, while 34% of women encountering delivery complications underwent emergency C-sections. Lastly, the investigation explored the influence of ANC visits on C-section prevalence. The findings indicated that 68% of women completing more than four ANC visits opted for pre-planned C-sections, while 32% of women with over four ANC visits underwent emergency C-sections.

Overall, the results suggest that C-sections are more prevalent in certain subgroups of the population in Andhra Pradesh and that factors such as wealth quintile, birth order, and ANC visits may impact the likelihood of elective versus emergency C-section deliveries.

Table 4 presents the results of the t-test used to compare the mean prevalence of women who underwent C-section deliveries in private and public hospitals. The mean prevalence of C-section deliveries among women who opted for public health facilities was approximately 0.262, with a standard deviation of 0.44 and a 95% confidence interval of 0.239-0.285. On the other hand, women who chose private health facilities had a mean prevalence of C-section deliveries of about 0.61, with a standard deviation of 0.486 and a 95% confidence interval of 0.591-0.643. The combined mean prevalence of C-section deliveries for both public and private health facilities was around 0.43, with a standard deviation of 0.495 and a 95% confidence interval of 0.414-0.451.

**Table 4:** Summary statistics for women undergoing caesarean sections in public and private healthcare facilities in Andhra Pradesh, India, NFHS-5 (2019-21) diff = mean(public) - mean(private), t = -20.0014, p<0.0001 Ho: diff = 0, degrees of freedom = 2732, Ha: diff ! = 0, Pr(|T| > |t|) = 0.0000

Group	Observations	Mean	Std. Err.	Std. Dev.	95% CI	p-value
Public	1,421	0.2625	0.0117	0.44014	0.239-0.285	0.0001
Private	1,313	0.6169	0.0134	0.48633	0.591-0.643	0.0001
Combined	2,734	0.4327	0.0095	0.49554	0.414-0.451	
Difference		-0.3544	0.0177		-0.389 - -0.320	

The mean difference between the prevalence of C-section deliveries in private and public health facilities was calculated to be (-0.354), indicating that private health facilities had a significantly higher prevalence of C-section deliveries than public facilities. The means are comparable, and the test is significant, providing strong evidence that private health facilities had a significantly higher prevalence of C-section deliveries than public facilities.

Table 5 presents the results of logistic regression analysis that investigated the association between the type of C-section delivery and selected background characteristics, including birth order, maternal age during delivery, and birth spacing. The analysis aimed to identify predictors of elective or emergency C-section delivery.

**Table 5:** Logistic regression estimates of the type of caesarean section delivery by various background characteristics among women in Andhra Pradesh, India, NFHS-5 (2019-21) OR: Odds ratio; CI: Confidence interval; ®: Reference category *, **, and *** refer to < 0.05, < 0.01, and < 0.001 levels of significance, respectively.

Background characteristics	Model 1	Model 2	Model 3	Model 4
	AOR (95% CI)	AOR (95% CI)	AOR (95% CI)	AOR (95% CI)
Birth order				
1 ®2	0.62*** [0.55-0.71]			0.60*** [0.51-0.70]
3 and above	0.77 [0.60–1.01]			0.76 [0.58–1.00]
Maternal age during delivery				
20 years and below ®21 to 30 years		0.82 [0.66–1.40]		0.84 [0.46–1.52]
31 years and above		0.93 [0.68–1.28]		0.87 [0.45–1.65]
Birth spacing				
Less than 2 years ®, 2 to 3 years			0.84 [0.64–1.10]	0.83 [0.64–1.09]
More than 3 years			0.83 [0.65-1.06]	0.82 [0.63–1.06]
Constant	0.67 [0.60-0.74]	0.50 [0.39–0.65]	0.47 [0.40- 0.55]	0.70 [0.37–1.34]

Model 1 showed that, compared to women with birth order one, women with birth order two had 38% lower odds of undergoing an emergency C-section delivery (AOR: 0.62***, 95%CI: 0.55-0.71). Similarly, women with birth order three and above had 23% lower odds of having an emergency C-section delivery. These results suggest that birth order is an important predictor of the type of C-section delivery, with women who have had more previous births being less likely to have an emergency C-section delivery.

In Model 2, maternal age was considered as a predictor, and it was found that women aged 21 and above had 18% lower odds of having an emergency C-section delivery as compared to women who were less than 20 years old at the time of delivery. This finding suggests that older women are less likely to require emergency C-section delivery.

Model 3 included birth spacing as a predictor, and the analysis revealed that women with a birth spacing of two to three years had 16% lower odds of having an emergency C-section delivery than women with less than two years of birth spacing. This finding implies that longer birth spacing may reduce the risk of emergency C-section delivery.

In Model 4, all three background characteristics were considered simultaneously, and it was found that only one subgroup of birth order was significant. The overall model was not significant, suggesting that the combination of birth order, maternal age, and birth spacing did not significantly predict the type of C-section delivery.

## Discussion

The rising prevalence of C-section deliveries is a significant health issue for women, with studies indicating that repeat C-sections are increasingly common in hospitals [1]. Our study has pinpointed various significant factors associated with elevated C-section rates in Andhra Pradesh. These factors encompass age, educational attainment, residential location, economic status, parity, and the location of delivery. Notably, age emerges as a crucial determinant influencing the probability of undergoing a C-section, with women exhibiting higher educational levels and opting for delayed marriage and childbirth showing a greater likelihood of undergoing this medical procedure.

In urban areas, a variety of factors can influence the frequency of C-section deliveries. Among all the factors, the place of delivery - whether a private or public medical institution - is becoming increasingly influential in determining the likelihood of a C-section delivery. Researchers have found strong correlations between socio-economic and cultural factors and the rising incidence of cesarean deliveries, with C-sections in private hospitals emerging as a primary driver of this trend [12-14]. The factors include the availability of advanced health facilities with state-of-the-art maternal health services, the tendency of women to opt for private medical facilities, high rates of maternal healthcare utilization, and competition for profit [15-20].

Over the past decade, the public-private gap in C-section deliveries has widened considerably, with the disparity between C-section delivery rates among younger and older mothers increasing dramatically. The rich-poor gap has also grown in overall C-section delivery rates, including voluntary procedures. Economic status appears to be one of the key factors contributing to the rising prevalence of C-section births [21-23].

The cesarean rates escalated from 4.4% in NFHS-1 (1992-93) to 40.1% in NFHS-4 (2015-16) and further increased to 42.44% in NFHS-5 (2019-21). This suggests that the C-section rates in the state almost doubled in the past decade.

As per the study findings, a stark divide exists in C-section rates between private and public hospitals, with private institutions witnessing a significantly higher percentage. This disparity is particularly pronounced in urban settings, where factors such as wealth, access to high-quality healthcare, and awareness likely contribute. While education emerges as a key influencer, especially in elective C-sections, financial limitations may restrict poorer households from accessing private hospitals and their higher C-section rates. However, the rising C-section trend in public hospitals hints at additional influences beyond affordability, potentially including evolving socio-economic realities, demographic shifts, health circumstances, and changing perceptions surrounding C-sections.

The data revealed that there was a significant difference in the prevalence of C-sections between public and private healthcare institutions in Andhra Pradesh. About 32% of women underwent C-sections in public hospitals, while the figure was much higher at 68% in private facilities. A substantial number of women across various districts in the state opted for private healthcare institutions for their C-section deliveries. Religion and place of delivery were also found to be important factors, as Christian women were more likely to choose public health facilities, while Muslim women were more likely to undergo C-sections in private hospitals. A statistical analysis revealed that the mean difference between C-section rates in private and public hospitals was -0.354, which was statistically significant. This result indicates that private facilities had a higher prevalence of C-section deliveries compared to public hospitals. Overall, the findings suggest that the choice of healthcare facility for C-section deliveries varies significantly based on several background characteristics such as religion, caste, wealth index, education, birth order, and complications during delivery.

The prevalence of elective C-sections was found to be relatively higher than emergency C-sections, with significant differentials observed between the two categories. Among women from the richest wealth quintile, almost one-third had planned C-sections, whereas half of the women from the poorest quintile had emergency C-sections. Additionally, among women who did not experience complications during delivery, one-third underwent elective C-sections. Birth order, maternal age during delivery, and birth spacing were significant predictors of the type of delivery, whether elective or emergency C-sections. However, when all three factors were considered together, only a specific subgroup of birth order was found to be significant. Thus, the overall model was not deemed significant.

The overall prevalence of elective C-section delivery in Andhra Pradesh indicates a higher prevalence of elective cases. Additionally, women in the C-section group had a higher relative risk of being underweight, having mild anaemia, experiencing prolonged labor, and vaginal bleeding after delivery.

## Conclusions

The study findings revealed a noteworthy rise in the occurrence of C-section deliveries in Andhra Pradesh. When considering different districts, the highest prevalence of C-section deliveries was identified in Krishna, Srikakulam, West Godavari, Prakasam, Guntur, and East Godavari districts of Andhra Pradesh. Notably, urban women exhibited the highest prevalence of C-section deliveries. Moreover, there was an escalating trend in the prevalence of C-section deliveries with an increase in both educational attainment and wealth quintile. Private healthcare facilities were the favored delivery location for approximately one-third of women in Andhra Pradesh.

The findings suggest that the prevalence of elective C-section deliveries was relatively higher across all districts in Andhra Pradesh than emergency C-section deliveries. This information could be beneficial in improving maternal healthcare services and identifying the districts requiring more attention in this regard. The results could be used to develop targeted interventions and policies aimed at reducing the prevalence of C-section deliveries, especially elective ones, and promoting safe vaginal deliveries, thereby improving maternal and neonatal health outcomes.
